# The effects of handedness and age on hand role selection in bimanual tasks

**DOI:** 10.1007/s00221-026-07301-1

**Published:** 2026-04-27

**Authors:** Harry T. Jordan, Milly G. Darragh, Cathy M. Stinear

**Affiliations:** Department of Medicine, University of Auckland, Private Bag 92019, Auckland, 1142 New Zealand

**Keywords:** Hand role selection, Ageing, Bimanual coordination, Asymmetric, Handedness

## Abstract

Most tasks performed in everyday life require both hands to perform asymmetric movements. Such tasks require one hand to act as a manipulator and the other hand as a stabiliser, and choosing the hand to perform each role is termed hand role selection. The dominant and non-dominant hand are usually spontaneously assigned as the manipulator and stabiliser, respectively. Both handedness and age are known to affect coordination during bimanual tasks, but their influence on spontaneous hand role selection across a range of tasks is unknown. This study investigated the effects of handedness and age on spontaneous hand role selection during routine bimanual tasks. Fifty healthy adults (mean age = 53, range 19 − 85 years) performed seven bimanual tasks with five repeats each. Participants were naïve to the study’s purpose to avoid any influence on their hand role selection. Handedness was determined using the 10-item Edinburgh Handedness Inventory. Hand role selection in each trial was noted to calculate a Preference score out of 35 for each participant. Overall, participants had a strong preference to consistently use their dominant hand as a manipulator across all tasks. Preference to use the dominant hand as a manipulator was task-dependent and correlated positively with handedness, but it did not correlate with age. These results show the degree of handedness, but not age, affects consistency of hand role selection across a range of bimanual task, and that preference to use the dominant hand as a manipulator varies between tasks.

**Supplementary Information:**

The online version contains supplementary material available at 10.1007/s00221-026-07301-1.

## Introduction

Everyday interactions with objects are often asymmetric bimanual tasks where each hand performs a distinct role as either the object manipulator or stabiliser (Leppanen et al. 2019; Woytowicz et al. 2018). The manipulator role typically requires finer motor skills and dexterity while the stabiliser role typically requires holding an object steady. For example, when opening a water bottle the manipulator will unscrew the lid while the stabiliser holds the bottle steady.

Hand role selection refers to assigning a hand as either the manipulator or stabiliser during a bimanual task (Guiard 1987). Previous studies have examined spontaneous hand role selection when adults perform naturalistic bimanual tasks with no instructions regarding hand role selection. People preferentially used their dominant hand as the manipulator and non-dominant hand as the stabiliser during a high-precision watch making task (Yao et al. 2021), when constructing models using blocks (Stone et al. 2013), or pouring water from a pitcher into a cup (Salters and Scharoun Benson 2022; Scharoun et al. 2016). These tasks have ecological validity, however each study only examined hand role selection for a single task. Leppanen et al. reported a strong preference to use the dominant hand as a manipulator across ten routine tasks, however only four tasks were bimanual (Leppanen et al. 2019). These observational findings are corroborated by a study that found participants self-report preferring to use their dominant and non-dominant hands as the manipulator and stabiliser, respectively, across 40 routine bimanual tasks (Salters & Scharoun Benson 2022). No studies to our knowledge have explored spontaneous hand role selection across a range of routine tasks.

Handedness refers to the consistent preference to use one hand over the other for a given task, and may affect hand role selection (Serrien et al. 2006). Handedness is often described in a binary manner with approximately 90% of the global population self-reporting as right-hand dominant and 10% as left-hand dominant (de Kovel et al. 2019; Peters et al. 2006; Vuoksimaa et al. 2009). Previous studies of hand role selection during bimanual tasks have described handedness in this typical binary manner (Salters and Scharoun Benson 2022; Scharoun et al. 2016; Stone et al. 2013; Yao et al. 2021). However, viewing handedness as a spectrum allows investigation into whether the strength of handedness, which is how strong the preference to use a hand for a task is, influences a person’s consistency of hand role selection for a given task, and whether hand role consistency varies across tasks. (Kraus 2023). For example, two people could self-report as right-handed but one is strongly right-handed and the other is weakly right-handed. A handedness spectrum also provides insight into ambidexterity, which is when a person has no hand use preference for any given task. Steenhuis investigated how self-reported handedness affected the spontaneous use of the dominant hand as a manipulator across at least 14 routine tasks, however the full list of tasks used was not reported and included a mixture of unimanual and bimanual tasks, the number of trials for each task was not reported, and the position of the items varied between trials which affects hand role selection (Salters and Scharoun Benson 2022; Scharoun et al. 2016; Steenhuis 1999). Leppanen et al. found a positive correlation between the strength of hand dominance and preference to use the dominant hand as a manipulator across ten routine tasks, however only four tasks were bimanual (Leppanen et al. 2019). It is currently unknown whether the strength of handedness influences the consistency of hand role selection across different routine bimanual tasks.

Another factor that may influence hand role selection in adulthood is age. A meta-analysis of 47 studies found that older adults are generally less accurate, more variable, and have longer movement times compared to younger adults across a range of bimanual tasks (Kang et al., 2022). One of the main theories underlying these findings is the Hemispheric Aging Reduction in Older Adults (HAROLD) model (Cabeza 2002; Kalisch et al. 2006). This model states that neural activity during cognitive tasks tends to be less lateralised in older adults when compared to younger adults, particularly in the prefrontal cortex (Cabeza 2002). The HAROLD model was developed regarding cognitive tasks but has since been extended to motor tasks. When performing unimanual tasks, older adults have greater activation of ipsilateral motor regions which leads to more symmetric activation of the primary motor areas of both hemispheres when compared to younger adults (Knights et al. 2021; Ward et al. 2008). Additionally, Wang et al. reported older adults showed increased symmetry in interlimb transfer of adaptation across the arms during a unimanual reaching task compared to younger adults, which further supports the HAROLD model (Wang et al. 2011). More symmetric cortical activity during unimanual tasks may also be related to the more similar performance by the dominant and non-dominant hands during unimanual tasks that is observed with aging (Kalisch et al. 2006; Przybyla et al. 2011; Sebastjan et al. 2017). Support for the HAROLD model also comes from bimanual motor tasks where older adults show increased activation in motor regions (Goble et al. 2010; Michels et al. 2018) and interhemispheric functional connectivity (Heitger et al. 2013) during simple rhythmic bimanual tasks compared to younger adults. It is possible that the greater symmetry in brain activation and increased interhemispheric connectivity could lead to less lateralized motor behaviour and more variable hand role selection in older adults than younger adults. Whether hand role selection during routine bimanual tasks is affected by age in adulthood has yet to be studied.

The aim of this study was to investigate how handedness and age influence spontaneous hand role selection in neurologically healthy adults performing seven routine bimanual tasks. The first hypothesis was that a positive correlation between the strength of handedness and the strength of hand role preference would be present. The second hypothesis was that a negative correlation between age and the strength of hand role preference would be present.

## Methods

### Participants

The inclusion criteria were to be at least 18 years old and not have any conditions, such as arthritis or tremors, that may affect hand and arm use. The planned sample size was a minimum of 40 participants as this was a novel evaluation with no prior data to inform a sample size calculation. All participants gave their written informed consent, and this study was approved by the institutional ethics committee. Participants were naïve to the study’s purpose since it may have influenced their hand role selections. Instead, participants were told the study was investigating the speed of performing routine bimanual tasks naturally so they should perform each task at their normal pace as they would in daily life. This report was prepared using the STROBE (Strengthening Reporting of Observational Studies in Epidemiology) guidelines (Supplemental materials) (von Elm et al. 2007).

### Edinburgh handedness inventory

Participants’ handedness was determined using the 10-item Edinburgh Handedness Inventory (EHI) which includes both unimanual and bimanual tasks (Oldfield 1971). Participants responded with one of five answers regarding which hand they preferentially use for each task: always left, usually left, either hand, usually right, or always right. The strength of handedness was determined using the laterality quotient (LQ): LQ = (R-L)/(R + L) × 100, with R and L representing the total scores for the right and left hands, respectively. A higher handedness score on the EHI (max of 100) indicates a stronger preference to use the right hand and a lower score (max of -100) indicates a stronger preference to use the left hand. For characterising handedness, participants with scores higher than 40 were categorised as being right-handed, participants with scores lower than -40 were categorised as being left-handed, and participants with scores from -40 to 40 were categorised as ambidextrous. For assessing hand role selection during bimanual tasks, participants with EHI scores above and below 0 were considered right-hand dominant and left-hand dominant, respectively. For statistical analyses, negative EHI values were converted to positive values to give an absolute EHI score which measures the strength of handedness regardless of whether it is right- or left-handedness (EHI_strength_). The EHI was completed at the end of the study after the bimanual tasks as it asks participants to actively consider their hand role selection in daily life and could have influenced spontaneous hand role selection.

### Bimanual tasks

Seven asymmetric bimanual tasks were selected with varying precision and force requirements to examine hand role selection. The tasks were familiar routine tasks requiring no specific prior skills. Participants were seated at a desk opposite the assessor. All objects were placed in the participant’s midline to ensure object position did not influence their hand role selection. Examples of how the task items were presented are shown in Figure I of the Supplemental Materials. Participants performed five trials for each of the seven tasks, for a total of 35 trials. The order of all 35 trials was randomised for each participant. Instructions provided to participants for each task are provided in Table 1 and were repeated for each trial.

**Table 1:** Hand roles and instructions for bimanual tasks

Task	Manipulator	Stabiliser	Instructions
Glue paper	Holds glue stick	Stabilises paper	“Apply one strip of glue onto this piece of paper”
Hammer nail	Holds hammer	Holds nail	“Hammer a nail into this piece of wood, hit the nail once”
Open jar	Twists lid	Holds jar	“Open this jar lid and place it on the table”
Peel carrot	Holds peeler	Holds carrot	“Peel one slice of this carrot using this vegetable peeler”
Sharpen pencil	Holds and rotates pencil	Holds sharpener	“Sharpen this pencil twice using the pencil sharpener”
Thread needle	Holds yarn	Holds needle	“Thread this length of yarn into this darning needle”
Zip pencil case	Pulls zip	Holds pencil case	“Zip up this pencil case”

The manipulator and stabiliser roles for each task are included in Table 1. The expected hand role selection for each task was to use the dominant hand as the manipulator and the non-dominant hand as the stabiliser. Each of the 35 trials were scored as a 1 or -1. A score of 1 indicated the expected hand role selection was used (dominant hand as manipulator) and a score of -1 indicates it was not used. Each task was performed five times and so a Preference score was calculated for each task which ranged from -5 to 5, with only odd numbered scores possible. Higher scores for an individual task indicated a stronger consistent preference to use the dominant hand as a manipulator. Scores from each task were summed together to provide an overall Preference score that assessed how often the expected hand role selection was used across all trials. Overall Preference scores ranged from -35 to 35, with a score of 35 indicating the participant used their dominant hand as a manipulator for all trials.

### Statistical analysis

Statistical analyses were performed using IBM SPSS version 29. Shapiro-Wilks tests confirmed all data were not normally distributed (all *p* < 0.01). Two-tailed Spearman’s rank correlations were conducted to examine the relationships between Preference scores and EHI_strength_, Preference scores and age, and EHI_strength_ and age. A Friedman test was performed with task-specific Preference scores ranging from -5 to 5 for each of the seven bimanual tasks to examine whether there was an effect of task on hand role selection. Post-hoc Wilcoxon signed-rank tests with Bonferroni corrections for multiple comparisons were used to explore any significant effect from the Friedman test. Values of *p* < 0.05 were considered statistically significant.

## Results

### Participants

Data were collected from 50 participants and their characteristics are provided in Table 2. The mean Preference score was 28 ± standard deviation (SD) 11, indicating that overall participants strongly preferred to use their dominant hand as a manipulator during the bimanual tasks.

**Table 2:** Demographic characteristics of participants (N = 50)

Sex (F/M)	33/17
Age (years)	53 ± 22 (19–85)
Handedness (R/L/Ambidextrous)	43/4/3
EHI_strength_	85 ± 23 (7–100)
Preference score (/35)	28 ± 11 (-15–35)

Values for continuous variables are means ± standard deviation, with range in parentheses. F, female; M, male; R, right-handed; L, left-handed; EHI, Edinburgh Handedness Inventory

### Age, handedness, and hand role selection

**Fig. 1:**
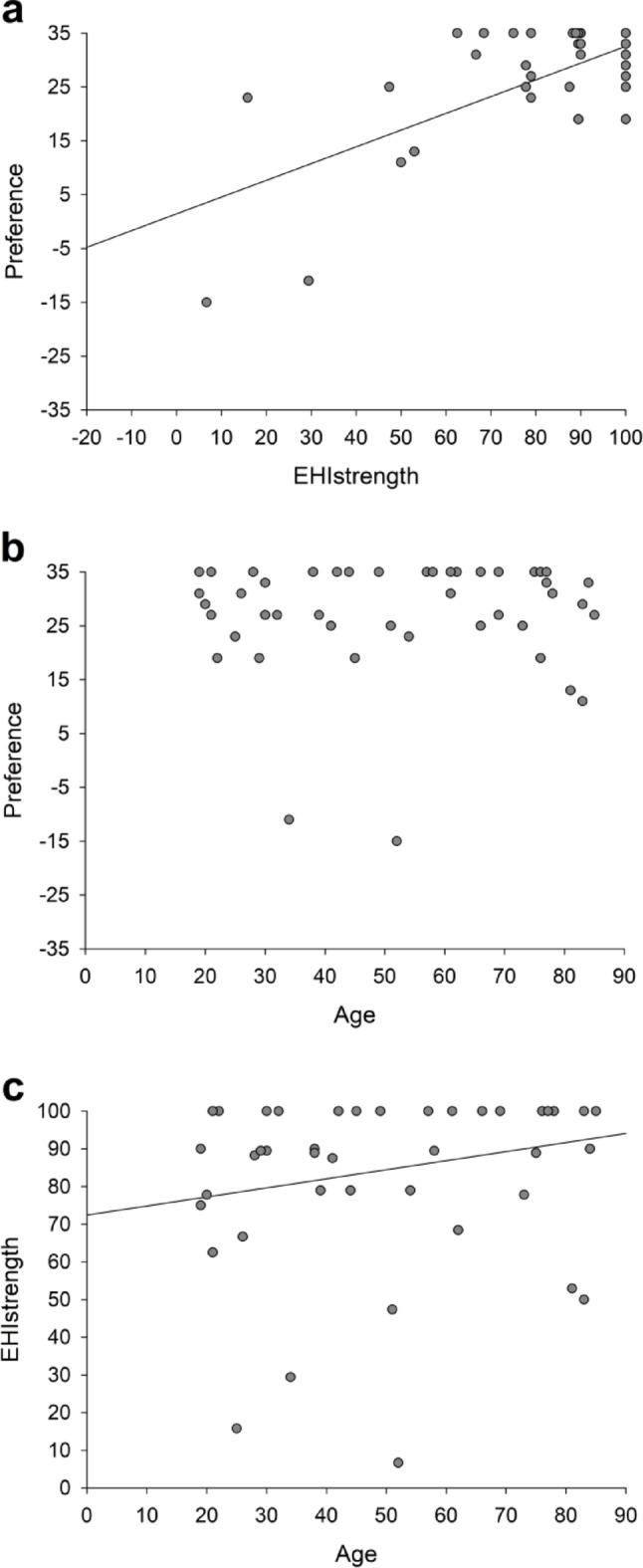
Correlations between Preference and EHIstrength (a), Preference and age (b), and age and EHIstrength (c). Significantcorrelations were found between Preference and EHIstrength as well as between age and EHIstrength. Each participant’s data is represented bya circle (N = 50)"

A significant positive correlation was observed between Preference and EHI_strength_ (p = 0.032, ρ = 0.30, Fig. 1A) and between age and EHI_strength_ (*p* = 0.017, ρ = 0.34, Fig. 1C). There was no significant correlation between Preference and age (p = 0.672, ρ = 0.06, Fig. 1B). Together these results indicate that participants with stronger handedness were more likely to use their dominant hand as a manipulator during bimanual tasks, and the strength of handedness increased with age.

### Preference between tasks

**Fig. 2:**
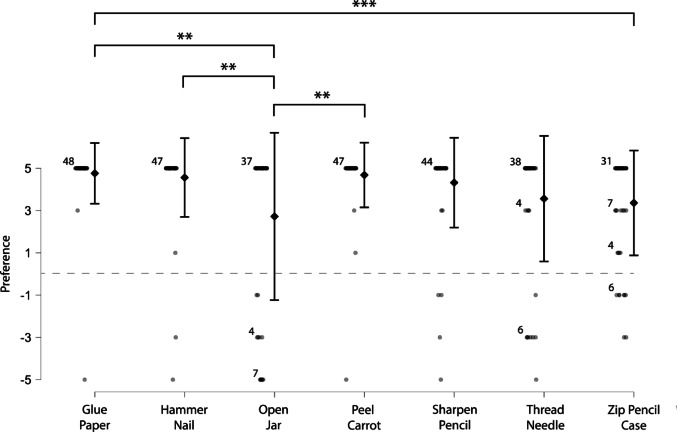
Preference scores for each of the seven bimanual tasks. Diamonds indicate mean values with error bars displayingstandard deviation. Circles indicate individual participant scores for each task and scores with more than 3 participants show the number of participants at that datapoint. Both ** and *** = p <0.01 (N = 50)"

A Friedman test revealed a main effect of task on Preference scores (p < 0.001, χ^2^ (6) = 38.3, Fig. 2). Post-hoc pairwise comparisons with Bonferroni corrections revealed that the mean Preference score was lower during the open jar task (mean ± SD = 2.72 ± 4.0) compared to the glue paper (*p* = 0.0016, *Z* = 3.16, mean ± SD = 4.76 ± 1.4), peel carrot (*p* = 0.0017, *Z* = 3.14, mean ± SD = 4.68 ± 1.5), and hammer nail tasks (*p* = 0.0023, *Z* = 3.04, mean ± SD = 4.56 ± 1.9). Mean preference was also lower during the zip pencil case task (mean ± SD = 3.36 ± 2.5) compared to the glue paper task (*p* = 0.0018, *Z* = 3.12). These results indicate the strength of participants’ preference to use their dominant hand as a manipulator was task-dependent.

## Discussion

The present study investigated the effects of handedness and age on spontaneous hand role selection across routine asymmetric bimanual tasks. In support of the first hypothesis, the strength of handedness positively correlated with Preference scores for hand role selection. The second hypothesis was not supported as there was no correlation between age and Preference scores for hand role selection. Furthermore, preference to use the dominant hand as a manipulator varied between tasks. Together these findings indicate that hand role selection is influenced by the strength of handedness and varies by task.

### Influence of handedness on hand role selection

There was a positive relationship between self-reported handedness and Preference scores. Participants with stronger hand dominance were more likely to spontaneously use their dominant hand as a manipulator across a range of routine tasks. Previous studies have also shown people spontaneously use their dominant hand as a manipulator, but they did not evaluate the strength of handedness (Salters and Scharoun Benson 2022; Scharoun et al. 2016; Stone et al. 2013; Yao et al. 2021). Leppanen et al. found a positive correlation between using the dominant hand as a manipulator and absolute EHI score (Leppanen et al. 2019). However, Leppanen et al. included six unimanual tasks and four bimanual ones with no separate analyses between task types, and only performed a single trial for each task. Regardless, the current study supports previous results showing participants prefer to spontaneously use their dominant hand as a manipulator.

The present results are consistent with the dynamic dominance hypothesis which proposes the dominant arm has specialised expertise for the predictive control of dynamics, whilst the non-dominant arm has specialised competency for positional stability in unpredictable environments (Sainburg 2005; Stone et al. 2013; Yao et al. 2021). Based on the dynamic dominance hypothesis, it is the specialised motor control aspects of the dominant and non-dominant upper limbs that lead to the spontaneous preference to use them as a manipulator and stabiliser, respectively. Studies have supported the dynamic dominance hypothesis across conditions in bimanual reaching tasks, younger and older adults, and left-handed subjects (Mutha et al. 2013; Scharoun et al. 2016). Further support comes from a task which had participants mimic bread slicing while both hands were physically connected with a spring. In agreement with the dynamic dominant hypothesis, the non-dominant hand demonstrated superior stabilisation compared to the dominant hand, but the dominant hand was superior at reaching (Woytowicz et al. 2020, 2018). The degree of hemispheric specialisation is greater in participants with stronger hand dominance and this may partially explain the current study’s finding that participants with stronger hand dominance were more likely to spontaneously use their dominant hand as a manipulator (McLagan et al. 2024; Woytowicz et al. 2018). This study demonstrates that stronger hand dominance is linked to a higher preference to use the dominant hand as a manipulator during a range of routine bimanual tasks.

### Influence of age on hand role selection

This is the first study to investigate the relationship between aging and hand role selection preference across a range of routine bimanual tasks. We hypothesised there would be a negative correlation between age and Preference scores based on the HAROLD model, which posits that neural activity during cognitive and motor tasks tends to be less lateralised in older adults compared to younger adults (Cabeza 2002). However, this hypothesis was not supported as no age-related reduction in hand role preference was observed during these routine tasks.

There are several potential explanations for why age did not influence hand role selection. One possibility is that the HAROLD model is not applicable behaviourally during routine bimanual tasks. Support for the HAROLD model with motor control has come from fMRI (Knights et al. 2021; Ward et al. 2008) and behavioural studies (Kalisch et al. 2006; Przybyla et al. 2011; Sebastjan et al. 2017; Wang et al. 2011) using novel unimanual motor tasks, as well as fMRI studies using rhythmic bimanual tasks (Goble et al. 2010; Heitger et al. 2013; Wang et al. 2011), in contrast to the routine bimanual tasks of the current study requiring discrete movements. Another potential reason is that age was analysed as a continuous variable, which was done to include adults across the full age spectrum. Previous studies showing detrimental effects of aging on bimanual performance or coordination typically group participants into discrete age brackets, and often with at least 20 year differences between groups (Gulde et al. 2019; Kalisch et al. 2006; Kang et al., 2022; Przybyla et al. 2011; Wishart et al. 2000). It is also possible that the tasks performed in this study were routine and too simple to elicit any age-related changes in hand role selection preference. Previous studies of bimanual performance and coordination with aging have found older adults do worse than younger adults during difficult but not simple versions of the same task (Gulde et al. 2019; Wishart et al. 2000). A separate study found interhemispheric connectivity was stronger in older adults performing a rhythmic task asymmetrically compared to a simpler symmetric condition, but this difference wasn’t present in younger adults (Heitger et al. 2013). Moreover, a study found the specialised roles of the dominant and non-dominant limbs as manipulator and stabiliser, as predicted by the dynamic dominance hypothesis, decreased with age during difficult unimanual tasks (Przybyla et al. 2011). In contrast, a separate study found the specialised roles of the dominant and non-dominant limbs was higher in older adults than younger adults during a virtual reality bimanual task imitating bread slicing (Woytowicz et al. 2020). Although the bread slicing task used a physical spring to constrain the arms, the task involved reaching in a virtual reality space with restrictions to distal upper limb joint and trunk movements. These proposed explanations are not mutually exclusive and may each have contributed to the study’s finding that age in adulthood does not affect hand role selection across routine bimanual tasks.

### Task-specificity of hand role selection

This study used bimanual tasks that are relevant to everyday life and require no specific skills to complete. Several previous studies on hand role selection have focussed on tasks that were more physically or mentally demanding (Stone et al. 2013; Yao et al. 2021) than the tasks in this study which were deliberately easy and familiar. Other studies have also used easy and familiar tasks but with replacements made to the task materials. For example, Leppanen et al. used a series of unimanual and bimanual tasks including a hammering task where participants used a rubber-tipped hammer to strike putty rather than a nail (Leppanen et al. 2019). Salters et al. had participants pick up a jug and glass to imitate pouring without any liquid (Salters and Scharoun Benson 2022), although a similar study actually had participants pour liquid (Scharoun et al. 2016). Using routine tasks with ecologically relevant objects means the current study’s hand role selections were spontaneous and the results have real-world applications.

Hand role preference was affected by task. All seven tasks were asymmetric. Five of the tasks involve using two objects to achieve a goal: glue paper, hammer nail, peel carrot, sharpen pencil, and thread needle. The two remaining tasks, open a jar and zip a pencil case, involve both hands interacting with a single object to achieve a goal. Less spatial coordination between the hands is needed for tasks requiring one object compared to two objects, which may have contributed to hand role preference being weaker for the open jar and zip pencil case tasks compared to several of the other tasks with two objects. Additionally, there is evidence that motor control and interhemispheric demands are greater when both hands are interacting with the same object compared to different objects either virtually (Duque et al. 2010; Mutha and Sainburg 2009) or with real items (Altermatt et al. 2023; Dietz et al. 2015). The current study’s results indicate that hand role preference differs by bimanual tasks, which could potentially be due to whether the task includes one or two objects. No studies have directly compared hand role selection in asymmetric tasks with one common-goal objective using one versus two objects, but this could be explored in the future.

### Strengths, limitations, and future directions

This study had several strengths. Objects for each task were presented in the midline of participants to minimise the influence of object placement on hand role selection (Bryden and Huszczynski 2011; Salters and Scharoun Benson 2022; Scharoun et al. 2016). A wide range of everyday tasks was used with different force and precision requirements. Furthermore, participants were unaware of the purpose of the study and completed the bimanual tasks prior to the handedness questionnaire, encouraging true spontaneous task completion.

This study also had several limitations. One is that it is not certain whether the current study’s results apply to all left-handed or ambidextrous participants because only four left-handed participant and three ambidextrous participants were included. Additionally, the total EHI score cannot distinguish between people with a strong preference to do some tasks with the right hand and other tasks with the left hand, which is sometimes referred to as mixed-handedness, and ambidextrous people who have no preference for hand use across all tasks. Similarly, the overall Preference measure used for hand role selection in the current study cannot distinguish between participants with a strong preference to use their dominant hand as a manipulator for some tasks and a stabiliser in other tasks versus participants with no strong preference for all tasks.

This study provides multiple directions for further research. First, it could be investigated whether hand role selection during bimanual tasks differs between individuals with mixed-handedness versus consistent handedness (Prichard et al. 2013). Second, the effect of task novelty on hand role selection could be explored as this study used routine tasks most people would be familiar with. The distribution of left- and right-handed people differs between cultural groups, but hand role selection across cultural groups is yet to be studied (Perelle and Ehrman 2005). The relationship between bimanual coordination and hand role selection could also be further explored, especially as less effective bimanual coordination has been reported in older adults than younger adults (Gulde et al. 2019; Kang et al. 2022).

## Conclusion

This study provides insight into spontaneous hand role selection across a range of bimanual tasks. These results show a significant positive relationship between the strength of handedness and the use of the dominant hand as the manipulator in routine bimanual tasks. This study indicates that spontaneous hand role selection can be measured using Preference scores, and that the preference to use the dominant hand as a manipulator varies between tasks.

## Supplementary Information

Below is the link to the electronic supplementary material.

Supplementary Material 1

## Data Availability

Data used in this study are available from the corresponding author upon reasonable request.

## References

[CR1] Altermatt M, Jordan H, Ho K, Byblow WD (2023) Modulation of ipsilateral motor evoked potentials during bimanual coordination tasks. Front Hum Neurosci 17:1219112. 10.3389/fnhum.2023.1219112 10.3389/fnhum.2023.1219112 PMC10509758 37736146

[CR2] Bryden PJ, Huszczynski J (2011) Under what conditions will right-handers use their left hand? The effects of object orientation, object location, arm position, and task complexity in preferential reaching. Laterality 16(6):722–736. 10.1080/1357650X.2010.514344 10.1080/1357650X.2010.514344 21391107

[CR3] Cabeza R (2002) Hemispheric asymmetry reduction in older adults: the HAROLD model. Psychol Aging 17(1):85–100. 10.1037/0882-7974.17.1.85 10.1037//0882-7974.17.1.85 11931290

[CR4] de Kovel CGF, Carrion-Castillo A, Francks C (2019) A large-scale population study of early life factors influencing left-handedness. Sci Rep 9(1):584. 10.1038/s41598-018-37423-8 10.1038/s41598-018-37423-8 PMC6345846 30679750

[CR5] Dietz V, Macauda G, Schrafl-Altermatt M, Wirz M, Kloter E, Michels L (2015) Neural coupling of cooperative hand movements: a reflex and fMRI study. Cereb Cortex 25(4):948–958. 10.1093/cercor/bht285 10.1093/cercor/bht285 24122137

[CR6] Duque J, Davare M, Delaunay L, Jacob B, Saur R, Hummel F, Hermoye L, Rossion B, Olivier E (2010) Monitoring coordination during bimanual movements: where is the mastermind? J Cogn Neurosci 22(3):526–542. 10.1162/jocn.2009.21213 10.1162/jocn.2009.21213 19309295

[CR7] Goble DJ, Coxon JP, Van Impe A, De Vos J, Wenderoth N, Swinnen SP (2010) The neural control of bimanual movements in the elderly: brain regions exhibiting age-related increases in activity, frequency-induced neural modulation, and task-specific compensatory recruitment. Hum Brain Mapp 31(8):1281–1295. 10.1002/hbm.20943 10.1002/hbm.20943 PMC6871108 20082331

[CR8] Guiard Y (1987) Asymmetric division of labor in human skilled bimanual action: the kinematic chain as a model. J Mot Behav 19(4):486–517. 10.1080/00222895.1987.10735426 10.1080/00222895.1987.10735426 15136274

[CR9] Gulde P, Schmidle S, Aumuller A, Hermsdorfer J (2019) The effects of speed of execution on upper-limb kinematics in activities of daily living with respect to age. Exp Brain Res 237(6):1383–1395. 10.1007/s00221-019-05507-0 10.1007/s00221-019-05507-0 30887078

[CR10] Heitger MH, Goble DJ, Dhollander T, Dupont P, Caeyenberghs K, Leemans A, Sunaert S, Swinnen SP (2013) Bimanual motor coordination in older adults is associated with increased functional brain connectivity—a graph-theoretical analysis. PLoS ONE 8(4):e62133. 10.1371/journal.pone.0062133 10.1371/journal.pone.0062133 PMC3639273 23637982

[CR11] Kalisch T, Wilimzig C, Kleibel N, Tegenthoff M, Dinse HR (2006) Age-related attenuation of dominant hand superiority. PLoS ONE 1(1):e90. 10.1371/journal.pone.0000090 10.1371/journal.pone.0000090 PMC1762407 17183722

[CR12] Kang N, Ko DK, Cauraugh JH (2022) Bimanual motor impairments in older adults: an updated systematic review and meta-analysis. EXCLI J 21:1068–1083. 10.17179/excli2022-5236 10.17179/excli2022-5236 PMC9650695 36381648

[CR13] Knights E, Morcom AM, Henson RN (2021) Does hemispheric asymmetry reduction in older adults in motor cortex reflect compensation? J Neurosci 41(45):9361–9373. 10.1523/JNEUROSCI.1111-21.2021 10.1523/JNEUROSCI.1111-21.2021 PMC8580140 34580164

[CR14] Kraus E (2023) Beyond left and right handedness: a practice-based approach to assessing and analysing handedness dimensions and types. Springer, Berlin

[CR15] Leppanen ML, Lyle KB, Edlin FM, Schafke VD (2019) Is self-report a valid measure of unimanual object-based task performance? Laterality 24(5):538–558. 10.1080/1357650X.2018.1550493 10.1080/1357650X.2018.1550493 30468107

[CR16] McLagan B, Dexheimer J, Strock N, Goldstein S, Guzman S, Erceg D, Schroeder ET (2024) The role of transcutaneous electrical nerve stimulation for menstrual pain relief: a randomized control trial. Womens Health 20:17455057241266455. 10.1177/17455057241266455 10.1177/17455057241266455 PMC11282568 39066557

[CR17] Michels L, Dietz V, Schattin A, Schrafl-Altermatt M (2018) Neuroplastic changes in older adults performing cooperative hand movements. Front Hum Neurosci 12:488. 10.3389/fnhum.2018.00488 10.3389/fnhum.2018.00488 PMC6300783 30618675

[CR18] Mutha PK, Sainburg RL (2009) Shared bimanual tasks elicit bimanual reflexes during movement. J Neurophysiol 102(6):3142–3155. 10.1152/jn.91335.2008 10.1152/jn.91335.2008 PMC2804413 19793874

[CR19] Mutha PK, Haaland KY, Sainburg RL (2013) Rethinking motor lateralization: specialized but complementary mechanisms for motor control of each arm. PLoS ONE 8(3):e58582. 10.1371/journal.pone.0058582 10.1371/journal.pone.0058582 PMC3589347 23472210

[CR20] Oldfield RC (1971) The assessment and analysis of handedness: the Edinburgh inventory. Neuropsychologia 9(1):97–113 10.1016/0028-3932(71)90067-4 5146491

[CR21] Perelle IB, Ehrman L (2005) On the other hand. Behav Genet 35(3):343–350. 10.1007/s10519-005-3226-z 10.1007/s10519-005-3226-z 15864449

[CR22] Peters M, Reimers S, Manning JT (2006) Hand preference for writing and associations with selected demographic and behavioral variables in 255,100 subjects: the BBC internet study. Brain Cogn 62(2):177–189. 10.1016/j.bandc.2006.04.005 10.1016/j.bandc.2006.04.005 16797814

[CR23] Prichard E, Propper RE, Christman SD (2013) Degree of handedness, but not direction, is a systematic predictor of cognitive performance. Front Psychol 4:9. 10.3389/fpsyg.2013.00009 10.3389/fpsyg.2013.00009 PMC3560368 23386836

[CR24] Przybyla A, Haaland KY, Bagesteiro LB, Sainburg RL (2011) Motor asymmetry reduction in older adults. Neurosci Lett 489(2):99–104. 10.1016/j.neulet.2010.11.074 10.1016/j.neulet.2010.11.074 PMC3422634 21144883

[CR25] Sainburg RL (2005) Handedness: differential specializations for control of trajectory and position. Exerc Sport Sci Rev 33(4):206–213. 10.1097/00003677-200510000-00010 10.1097/00003677-200510000-00010 PMC10709818 16239839

[CR26] Salters D, Scharoun Benson SM (2022) Hand preference for unimanual and bimanual tasks: evidence from questionnaires and preferential reaching. Laterality 27(3):308–323. 10.1080/1357650X.2021.1990313 10.1080/1357650X.2021.1990313 34658296

[CR27] Scharoun SM, Scanlan KA, Bryden PJ (2016) Hand and grasp selection in a preferential reaching task: the effects of object location, orientation, and task intention. Front Psychol 7:360. 10.3389/fpsyg.2016.00360 10.3389/fpsyg.2016.00360 PMC4793775 27014165

[CR28] Sebastjan A, Skrzek A, Ignasiak Z, Slawinska T (2017) Age-related changes in hand dominance and functional asymmetry in older adults. PLoS ONE 12(5):e0177845. 10.1371/journal.pone.0177845 10.1371/journal.pone.0177845 PMC5448747 28558047

[CR29] Serrien DJ, Ivry RB, Swinnen SP (2006) Dynamics of hemispheric specialization and integration in the context of motor control. Nat Rev Neurosci 7(2):160–166. 10.1038/nrn1849 10.1038/nrn1849 16429125

[CR30] Steenhuis RE (1999) The relation between hand preference and hand performance: what you get depends on what you measure. Laterality 4(1):3–26. 10.1080/713754324 10.1080/713754324 15513101

[CR31] Stone KD, Bryant DC, Gonzalez CL (2013) Hand use for grasping in a bimanual task: evidence for different roles? Exp Brain Res 224(3):455–467. 10.1007/s00221-012-3325-z 10.1007/s00221-012-3325-z 23161156

[CR32] von Elm E, Altman DG, Egger M, Pocock SJ, Gotzsche PC, Vandenbroucke JP, Initiative S (2007) The strengthening the reporting of observational studies in epidemiology (STROBE) statement: guidelines for reporting observational studies. Ann Intern Med 147(8):573–577. 10.7326/0003-4819-147-8-200710160-00010 10.7326/0003-4819-147-8-200710160-00010 17938396

[CR33] Vuoksimaa E, Koskenvuo M, Rose RJ, Kaprio J (2009) Origins of handedness: a nationwide study of 30,161 adults. Neuropsychologia 47(5):1294–1301. 10.1016/j.neuropsychologia.2009.01.007 10.1016/j.neuropsychologia.2009.01.007 PMC2680751 19428393

[CR34] Wang J, Przybyla A, Wuebbenhorst K, Haaland KY, Sainburg RL (2011) Aging reduces asymmetries in interlimb transfer of visuomotor adaptation. Exp Brain Res 210(2):283–290. 10.1007/s00221-011-2631-1 10.1007/s00221-011-2631-1 PMC3116897 21424842

[CR35] Ward NS, Swayne OB, Newton JM (2008) Age-dependent changes in the neural correlates of force modulation: an fMRI study. Neurobiol Aging 29(9):1434–1446. 10.1016/j.neurobiolaging.2007.04.017 10.1016/j.neurobiolaging.2007.04.017 PMC2568861 17566608

[CR36] Wishart LR, Lee TD, Murdoch JE, Hodges NJ (2000) Effects of aging on automatic and effortful processes in bimanual coordination. J Gerontol B Psychol Sci Soc Sci 55(2):P85-94 10.1093/geronb/55.2.p85 10794187

[CR37] Woytowicz EJ, Westlake KP, Whitall J, Sainburg RL (2018) Handedness results from complementary hemispheric dominance, not global hemispheric dominance: evidence from mechanically coupled bilateral movements. J Neurophysiol 120(2):729–740. 10.1152/jn.00878.2017 10.1152/jn.00878.2017 PMC7132323 29742023

[CR38] Woytowicz EJ, Sainburg RL, Westlake KP, Whitall J (2020) Competition for limited neural resources in older adults leads to greater asymmetry of bilateral movements than in young adults. J Neurophysiol 123(4):1295–1304. 10.1152/jn.00405.2019 10.1152/jn.00405.2019 PMC7191525 31913762

[CR39] Yao K, Sternad D, Billard A (2021) Hand pose selection in a bimanual fine-manipulation task. J Neurophysiol. 10.1152/jn.00635.2020 10.1152/jn.00635.2020 PMC8325606 34107225

